# Parallel convolutional neural network and empirical mode decomposition for high accuracy in motor imagery EEG signal classification

**DOI:** 10.1371/journal.pone.0311942

**Published:** 2025-01-16

**Authors:** Jaipriya D., Sriharipriya K. C.

**Affiliations:** School of Electronics Engineering (SENSE), Vellore Institute of Technology, Vellore, Tamil Nadu, India; American University of the Middle East, KUWAIT

## Abstract

In recent years, the utilization of motor imagery (MI) signals derived from electroencephalography (EEG) has shown promising applications in controlling various devices such as wheelchairs, assistive technologies, and driverless vehicles. However, decoding EEG signals poses significant challenges due to their complexity, dynamic nature, and low signal-to-noise ratio (SNR). Traditional EEG pattern recognition algorithms typically involve two key steps: feature extraction and feature classification, both crucial for accurate operation. In this work, we propose a novel method that addresses these challenges by employing empirical mode decomposition (EMD) for feature extraction and a parallel convolutional neural network (PCNN) for feature classification. This approach aims to mitigate non-stationary issues, improve performance speed, and enhance classification accuracy. We validate the effectiveness of our proposed method using datasets from the BCI competition IV, specifically datasets 2a and 2b, which contain motor imagery EEG signals. Our method focuses on identifying two- and four-class motor imagery EEG signal classifications. Additionally, we introduce a transfer learning technique to fine-tune the model for individual subjects, leveraging important features extracted from a group dataset. Our results demonstrate that the proposed EMD-PCNN method outperforms existing approaches in terms of classification accuracy. We conduct both qualitative and quantitative analyses to evaluate our method. Qualitatively, we employ confusion matrices and various performance metrics such as specificity, sensitivity, precision, accuracy, recall, and f1-score. Quantitatively, we compare the classification accuracies of our method with those of existing approaches. Our findings highlight the superiority of the proposed EMD-PCNN method in accurately classifying motor imagery EEG signals. The enhanced performance and robustness of our method underscore its potential for broader applicability in real-world scenarios.

## 1. Introduction

The bioelectric information contained in electroencephalography (EEG) signals reflects the physiological processes taking place in the human brain [1–4]. They are frequently used with brain-computer interfaces (BCIs), which turn recorded brain activity into useful information to connect with outside surroundings. The brain-computer interface (BCI) is an innovative non-muscular communication system that connects directly from human intent to external equipment like computers, robots, unmanned aerial vehicles, and so on. BCI can assist individuals in directly controlling BCI-based robots to do a variety of activities through thought and has a very broad range of potential applications in daily life [5–7].

Numerous research projects use multiple electroencephalography (EEG) signal types, such as event-related potential (ERP), steady-state visually evoked potential (SSVEP), and motor imagery (MI). The event-related potential (ERP), known as P300 evoked potentials, demonstrates how sensitively a subject respond to stimuli [8–10]. By locating the SSVEP frequencies in the EEG data, steady state visually evoked potential (SSVEP), which has been employed in various applications including controlling the grasping of the robotic hand and evaluating adult visual acuity, can recognize a set of characters when visual stimulation is present. The feasibility of hybrid SSVEP + P300 visual BCI systems for quad-copter flight control, aiming to overcome limitations in existing BCI-based controls. The system integrates SSVEP and P300 potentials for enhanced control commands, facilitated by a GUI allowing users to control flight direction by gazing at visual stimuli buttons. Comparative analysis with conventional BCI and keyboard flight control systems demonstrates superior performance, though slightly lower than commercial keyboard controls. Importantly, the proposed system shows promise for aiding individuals with severe motor disabilities [11]. Additionally, motor imagery (MI) is another EEG signal that is frequently used. By converting MI frequencies into distinct commands, MI signals can control robot motion. EEG signals of two or more different types may merge to form a full signal.

One sort of EEG signal from the sensorimotor cortex is called the MI when a researcher imagines moving a body component without actually moving it. The physical parts of fictitious tasks could be the left and right hand, tongue, foot, fingers, shoulder, elbows, and so forth [12]. Thus, a crucial and essential issue in BCI research is the classification of various MI signal types. Brain activity, known as motor imagery (EEG), is observed when a participant intends to move limbs like their hands or feet. In response to these picturing or thinking tasks, the MI EEG signal is generated in the sensorimotor cortex portion of the brain. Researchers have used these MI signals to distinguish between various oscillatory brain activations for various tasks [13]. Utilizing several machine learning and deep learning methods, automated MI categorization has been carried out.

The motor-imagery brain state can be classified using the widely used modality of EEG, which uses a signal recording approach. The following are the challenges encountered during the classification of the motor imagery EEG signal. Although there are a lot of motor imagery tasks, training different binary classifiers takes a lot of time. Deep models are shown to require extensive training data and neural network attribute adjustments. During classification, the data loss is very high. For better performance, finding robust features is a challenging task, and, in most cases, there will be a higher failure rate. These difficulties with the traditional methods serve as motivation for developing a new way of categorizing the EEG data. This paper aims to develop a novel method to improve the classification of motor imagery EEG signals, and its contribution has been discussed below. The classification of MI EEG signals aims to improve the performance and accuracy of BCIs by effectively distinguishing between different motor imagery tasks. This would enable more reliable and efficient communication and control of external devices through brain activity. The classification of MI EEG signals contributes to advancements in EEG data processing and analysis techniques. Developing robust and efficient classification algorithms enhances the capabilities of neuroimaging technologies, paving the way for more sophisticated and accessible tools in clinical, research, and BCI applications.

The weakness and the limitations of the proposed work is the 1. Limited Robustness to Noise and Variability—Despite achieving high classification accuracy on the BCI competition IV datasets, the method’s robustness to noise and variability in real-world EEG recordings may be limited. EEG signals are inherently noisy, and variations in electrode placement, signal quality, and participant characteristics can affect classification performance. Future research could explore techniques to enhance the model’s robustness to such variability, such as data augmentation, denoising methods, or adaptive learning approaches -. 2. Transfer Learning Dependency The reliance on transfer learning to adapt the model to individual-specific datasets may introduce biases or limitations. Transfer learning assumes that features extracted from a group dataset are transferable to individual datasets, which may not always hold true due to inter-subject variability. Investigating alternative approaches or strategies to mitigate the need for transfer learning could improve the model’s generalizability and applicability. 3. Computational Complexity—The computational complexity associated with empirical mode decomposition and parallel convolutional neural networks may limit the model’s scalability and real-time applicability, especially in resource-constrained environments or applications requiring low-latency responses. Optimizing the model architecture, exploring light weight alternatives, or leveraging hardware acceleration techniques could help address computational challenges and improve efficiency.

**Fig 1:**
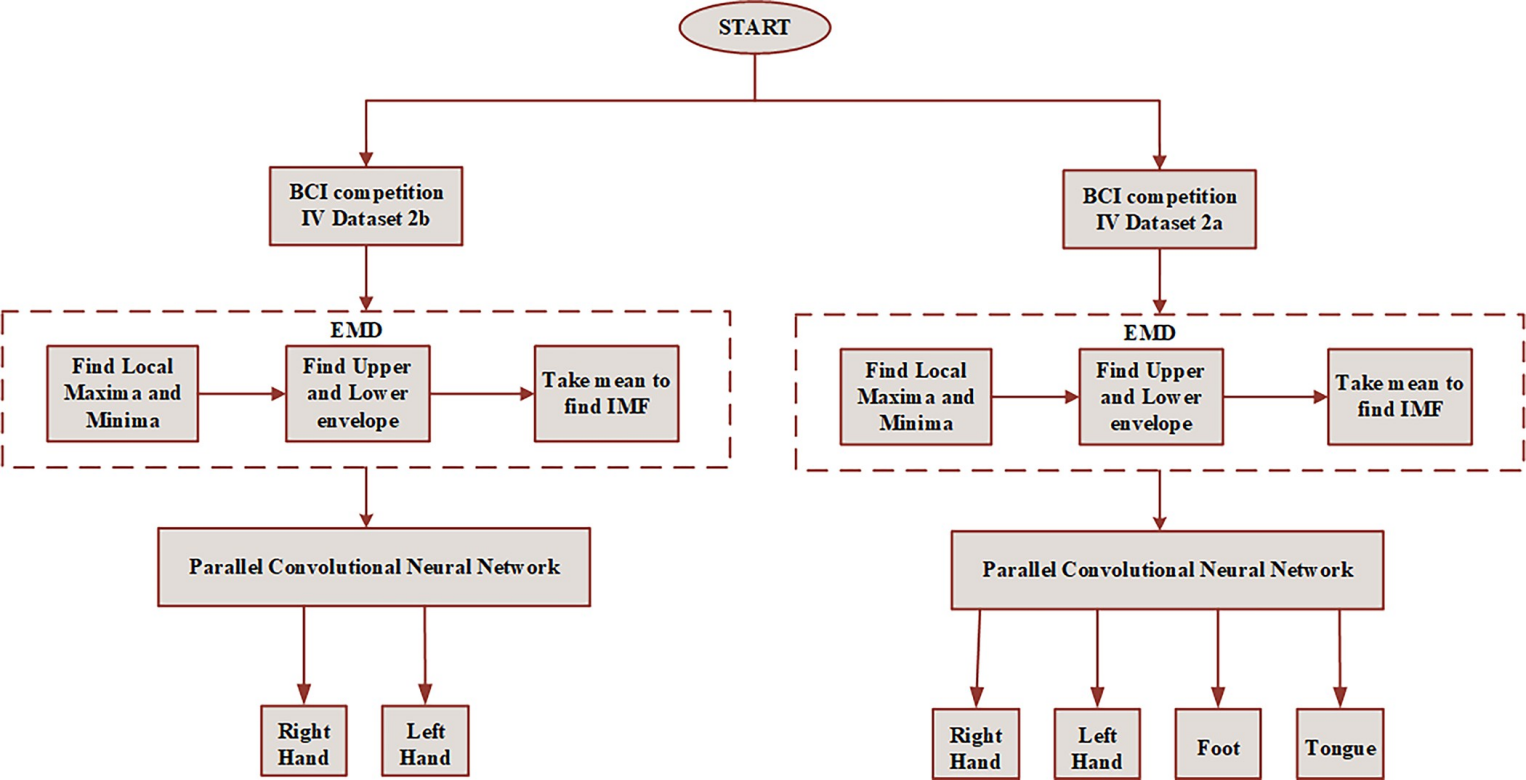
Overall block diagram of proposed EMD-PCNN work.

The rest of this paper is organized as follows: In Section 2, the related study of feature extraction and classification techniques used in motor imagery EEG signal analysis is discussed. In Section 3, the dataset description used in the proposed work, the details of the features of the EEG signal, and the basics of the empirical mode decomposition method and convolutional neural network algorithm for the EEG signal representation are described. The description of EMD-PCNN is proposed in Section 4. In Section 5, the classification results and the evaluation parameters are presented. Finally, we draw a conclusion in Section 6. The overall block diagram of the proposed work is illustrated in Fig 1.

## 2. Related study

**Fig 2:**
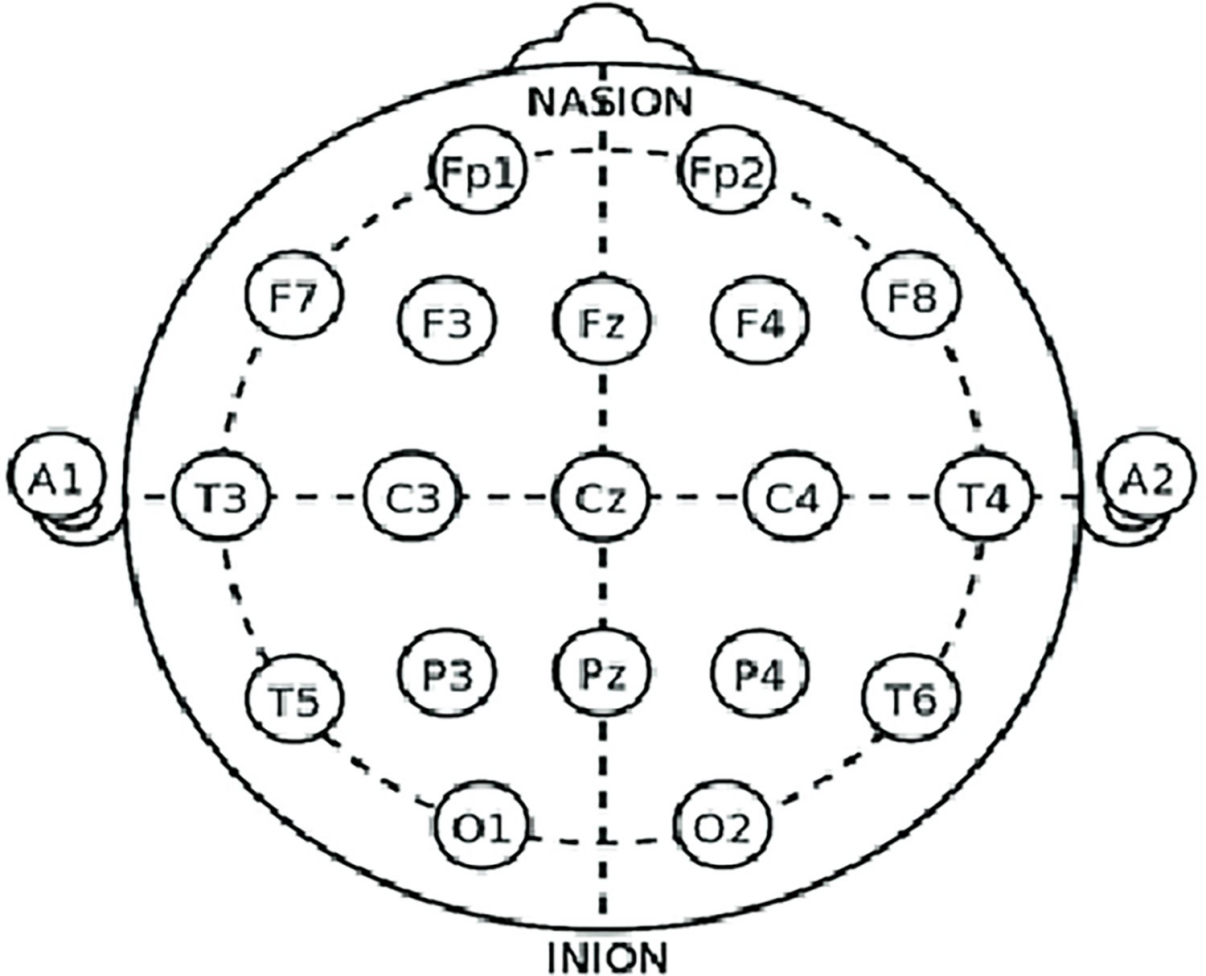
10–20 international system of EEG electrode placement.

The categorization of EEG signals generally involves four steps. The first phase is signal recording, where the EEG signals are recorded from willing individuals using the 10–20 international system, which is used as the accepted benchmark for scalp electrode location for EEG. The 10–20 international system of EEG electrode placement is illustrated in Fig 2.

Second, when the needed experimental sessions are completed by the subjects, the multichannel raw EEG data have a very low signal-to-noise ratio (SNR), requiring signal pre-processing to reduce the noise. Following pre-processing, the next stage is feature extraction, which is the process of extracting the usable features from the signals. Finally, classification is performed using these retrieved features, which is the last step. The methods available for feature extraction and classification are discussed in sections 2.1 and 2.2.

### 2.1 Feature extraction

EEG signals exhibit time-varying sensitivity, non-stationarity, and non-linearity. Numerous studies have identified mu and beta frequency bands as characteristics of motor imagery EEG signals because both real motions and imagined movements can cause changes in mu and beta rhythm. For example, the extraction of EEG-MI features makes extensive use of frequency decomposition techniques like the transform techniques DFT and FFT.

Feature extraction strategies were split into basic and advanced categories by Swati et al. [14]. The two fundamental methods were time domain and frequency domain, with frequency domain elements being more crucial for the detection of motor imagery. The two most sophisticated methods were space-time-frequency domain technology and time-frequency domain technology. Combining the features of the time and frequency domains, or even adding spatial properties, might enhance recognition ability. In addition to that, various wavelet techniques are also available for motor imagery EEG signals, such as discrete wavelet transform, wavelet packet transform, dual tree complex wavelet transform, etc. A well-known technique for extracting characteristics for motor imagery recognition is the common spatial pattern (CSP). The CSP approach was used to translate the multi-channel brain signals for particular activities converted into low-dimensional space for EEG categorization; such activities are the movements of the right and left hands [15].

### 2.2 Classification

There are various traditional algorithms available for the classification of motor imagery EEG signals. Among them, linear discriminant analysis and support vector machines are the most popular [16]. Additionally, the EEG-MI research employs the k-nearest neighbour, sparse Bayesian classifier, and fisher linear discriminant [17]. Furthermore, the neural network can adaptively extract data from the EEG and may integrate extracted features and pattern recognition into an entire model. Support vector machine (SVM) [18–20] and linear discriminant analysis (LDA) [21–25] have been the two most used classifiers in the field of EEG recognition during the last ten years. Nevertheless, one of the primary issues with traditional research was the low classification rate, which was caused by the signals’ low signal-to-noise ratio (SNR). Neural networks [26–28] and deep neural networks [29–33] are the classification algorithms that have been applied to the motor imaging brain computer interface.

Previous studies showed that academics from all sectors have embraced deep learning, particularly convoluted neural networks, which have been widely deployed in artificial intelligence, speech recognition, and other fields. However, research was still proceeding on CNN’s potential applications in the discipline of brain-computer interface. A deep convolution neural network model known as EEGNET was developed by Nayak et al. [34] and is capable of reliably differentiating EEG data from various BCI tasks. This result demonstrates that the EEGNET not only exhibited good cross-paradigm generalization capacity and strong robustness but also produced the same results as the most effective conventional EEG classification method, FBCSP. In order to train CNN for classification, Zapata et al. retrieved the original signal’s power spectral density (PSD) features [35].

Although the derived features only contain the EEG frequency domain characteristics, ignoring the time domain elements of EEG signals, the recognition rate was still greater than with the old method. The short-time Fourier transform (STFT) was used to turn the raw signals into time-frequency graphs, and the CNN was trained using the combined images from the C3 and C4 channels [36]. Spiking neural networks (SNNs) were introduced to the EEG classification challenge by Virgilio et al. [37], who were able to reach an accuracy of 0.83 for the binary classification of MI tasks. Third-generation artificial neural networks, or SNNs, are seen to have a significant chance of replacing second-generation ANNs like CNN [38].

The comparison of traditional ML algorithms with Deep Learning methods, focusing on Multi-Layered Perceptron (MLP) for Motor Imagery classification in BCIs. While Support Vector Machine (SVM) exhibits fast training and prediction, MLP achieves ≈90% accuracy in subject-independent classification with half the time, indicating its potential as a foundational framework for advanced BCI optimization. Further refinement could lead to highly accurate and robust subject-independent BCIs [39]. A multi-scale convolutional neural network (MS-CNN) for EEG-based motor imagery (MI) classification in brain-machine interfaces (BMIs), addressing challenges like inter-subject variability and low signal-to-noise ratio (SNR). By integrating discriminant user-specific features and employing data augmentation techniques, the model achieves superior classification accuracy and Cohen’s kappa-coefficient on the BCI competition IV2b dataset, outperforming existing EEG-based MI classification models. This framework holds promise for enhancing real-time human-robot interaction through efficient and robust EEG signal decoding [40].

A transfer learning-based multi-scale feature fused CNN (MSFFCNN) to address challenges in EEG-based motor imagery (MI) classification for brain-computer interfaces (BCIs). By offering four model variants including subject-independent and subject-adaptive configurations, the approach achieves high classification accuracy (94.06%) and kappa value (0.88) surpassing existing MI classification models, even with fewer training samples. The proposed framework presents an efficient solution for building robust MI-BCI systems, demonstrating the effectiveness of adaptive transfer learning in enhancing classification performance [41].

## 3. Materials and methods

### 3.1 Dataset description

We use two distinct motor imagery EEG datasets to assess our proposed algorithm in this research work to accurately measure its effectiveness. The size, number of channels, kind of motor imagery, and subject composition of these two datasets are very different from one another. The BCI Competition IV dataset 2a and the BCI Competition IV dataset 2b are publicly available datasets that contain motor imagery and EEG signal data and have been utilized by numerous researchers. We can thus easily compare our model with the findings of several other similar articles by using this dataset.

#### 3.1.1 BCI competition IV dataset 2a

**Fig 3:**
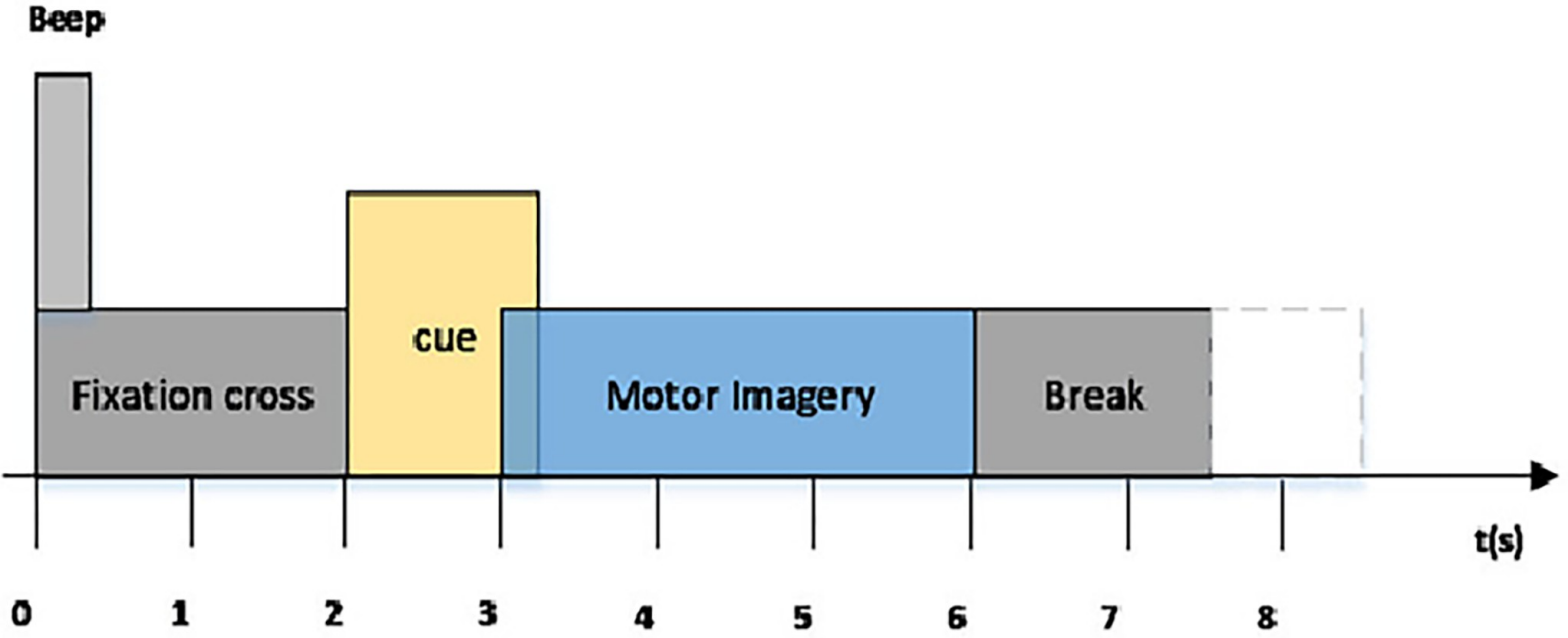
Timing diagram of the BCI competition IV dataset 2a paradigm.

A 22-channel motor imaging dataset was gathered for the BCI Competition IV and is called BCI Competition IV dataset 2a. Nine healthy individuals provided the four types of left hand, right hand, foot, and tongue imagery motions that make up this dataset. Consequently, the task is a four-class motor imagery EEG decoding challenge. This competition’s organizers offered a dataset that was band-pass filtered between 0.5 and 100 Hz and sampled at 250 Hz. A third-order Butterworth filter was used to band-pass filter the EEG signal between 4 and 38 Hz. For uniformity, an exponentially shifted panning window with a decay factor of 0.999 was applied to each electrode signal. The signals were then recorded, per previous study results, between 0.5 and 2.5 seconds after the initial occurrence of the motor imagery. The subjects completed the motor imagery task until the fixation cross disappeared from the screen at t = 6 s. No feedback was given. The timing diagram of the BCI competition IV dataset 2a has been depicted in Fig 3. The CNN input, or individual trial, in this instance has the form of (1, 22, 500). For the training and test sets, it appears that each individual ultimately produced 288 sets of EEG trials. In order to execute an early end during the first phase of the optimization process, the training set had been further separated into a validation set.

#### 3.1.2 BCI competition IV dataset 2b

**Fig 4:**
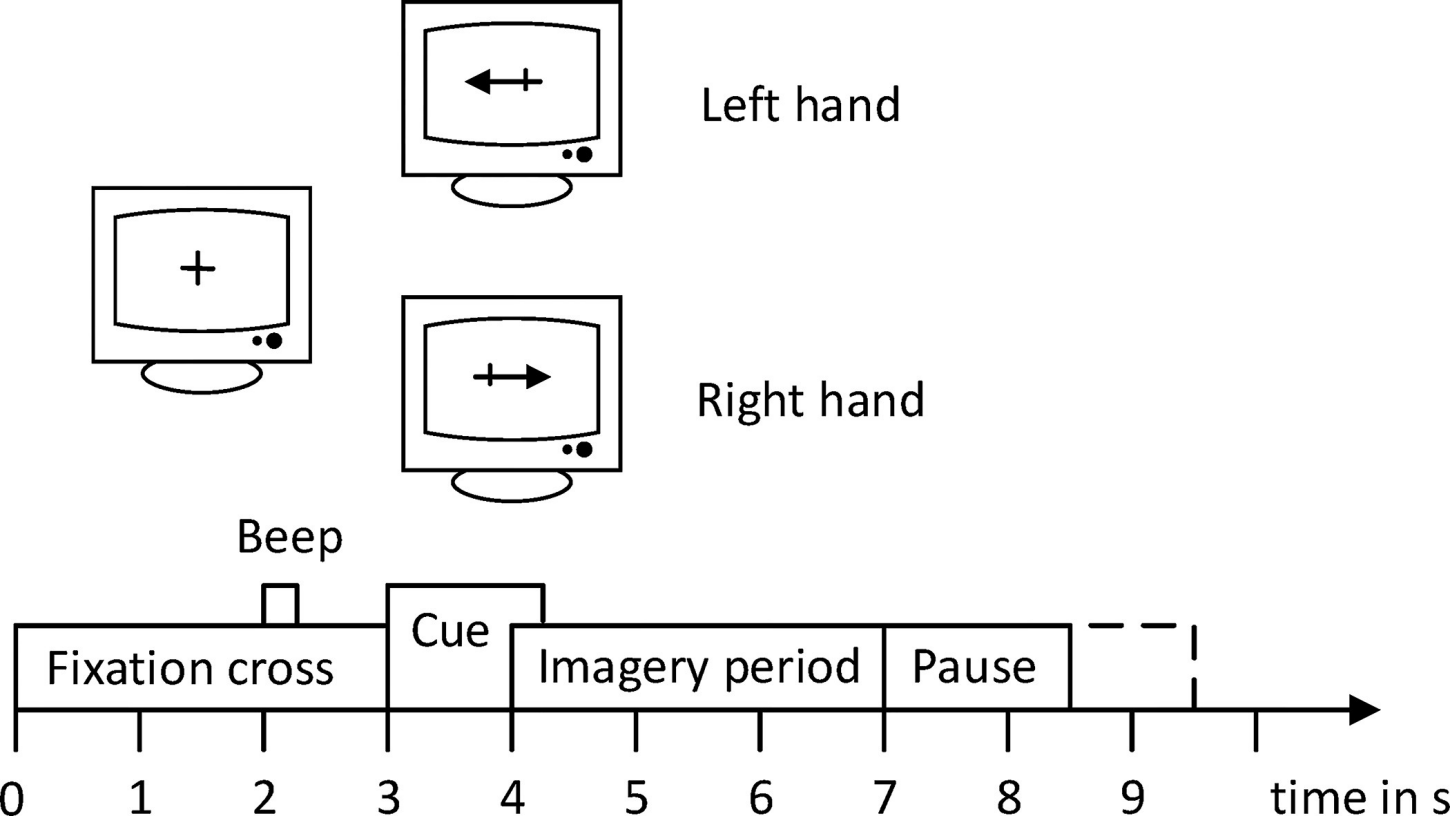
Timing diagram of BCI competition IV dataset 2b.

BCI Competition IV dataset 2b, a dataset is made up of nine EEG participants. There were five sessions available for every subject. The final set of recordings had feedback, but the first two contained training data without any. For each subject in this paper, only data without feedback has been chosen as the dataset. A fixed cross and an extra-brief auditory warning tone (1 kHz, 70 ms) were played at the beginning of each trial. A short while later, a visual indication emerged for 1.25 seconds, displaying an arrow pointing to the left or right, depending on the category that was requested. Within four seconds, the subjects had to picture the matching hand gestures. Every trial was separated by a minimum of 1.5 seconds of rest. To further prevent adaptation, the rest interval was extended by up to one second of randomization. The timing diagram of the BCI competition IV dataset 2b has been illustrated in Fig 4.

Three bipolar recordings (C3, Cz, and C4) were made at a sampling frequency of 250 Hz for the dataset. They had a 50 Hz trap filter turned on and were band-pass filtered between 0.5 Hz and 100 Hz. For every participant, the three bipolar recordings were located in slightly different areas. Experiments on the motor imagery task for both left- and right-handed motions were included in the data collection. There were 120 trials per session.

**Fig 5:**
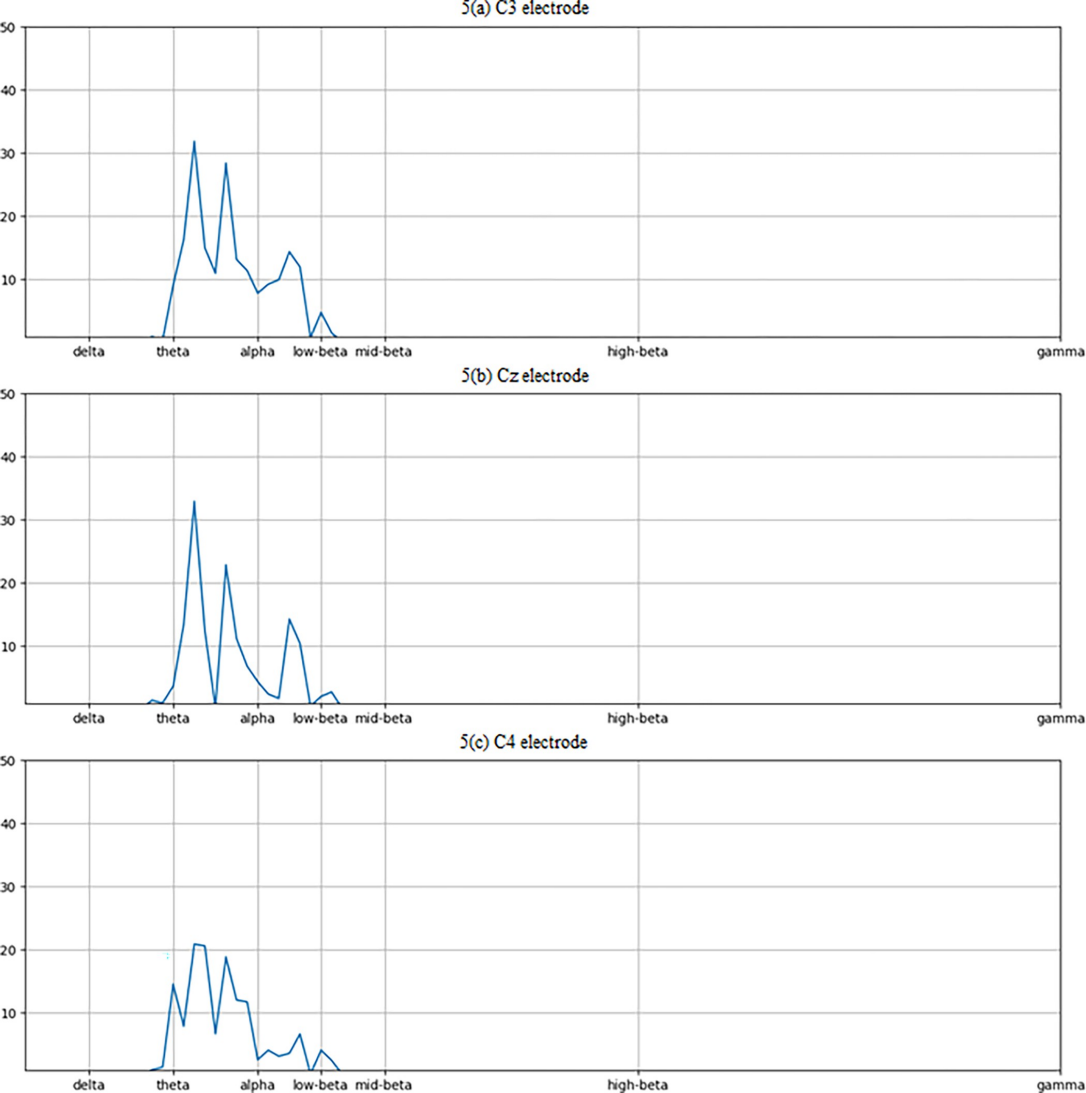
Diagram for Power Spectral Density of the electrodes (a) C3 electrode (b) Cz electrode (c) C4 electrode.

**Fig 6:**
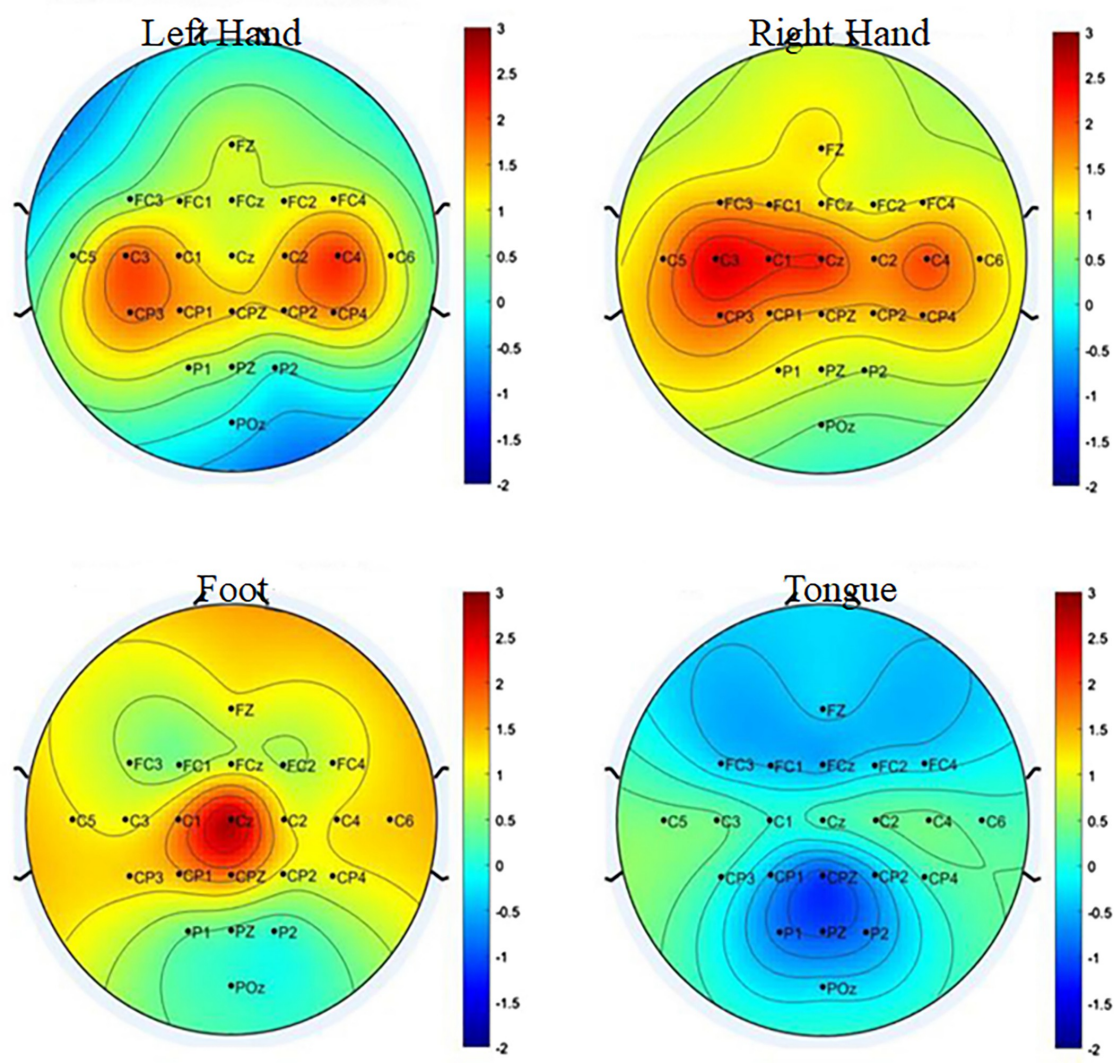
Diagram for the topographical maps of four classes (Right hand, Left hand, Foot, and Tongue).

The power spectral density (PSD) of C3, Cz, and C4 electrode is shown in Fig 5(A)–5(C). In motor imagery EEG signal classification, the power spectral density (PSD) of electrodes C3, C4, and CZ plays a crucial role in capturing frequency domain information associated with motor imagery tasks. C3, C4, and Cz electrodes are commonly selected for motor imagery EEG signal analysis due to their proximity to motor-related areas of the brain (central cortex). The PSD is typically across specific frequency bands relevant to motor imagery tasks, such as delta (1-4Hz), theta (4-8Hz), alpha (8-12Hz), beta (12-30Hz), and gamma (greater than 30Hz). These bands capture different neural oscillations associated with motor planning, execution, and inhibition. Overall, the PSD of electrodes C3, C4, and CZ provides valuable frequency domain information for discriminating between different motor imagery tasks, facilitating accurate classification in EEG-based brain-computer interface (BCI) systems. The topographical maps of four classes (Right hand, Left hand, Foot, and Tongue) is illustrated in Fig 6.

### 3.2 EMD

For nonlinear and nonstationary signals, empirical mode decomposition (EMD) is a fully data-driven technique for estimating time-frequency with highly localised information [42], by which the signal at hand is divided into a limited number of intrinsic mode functions (IMFs) and the intrinsic mode functions are defined as the components of AM/FM.

**Fig 7:**
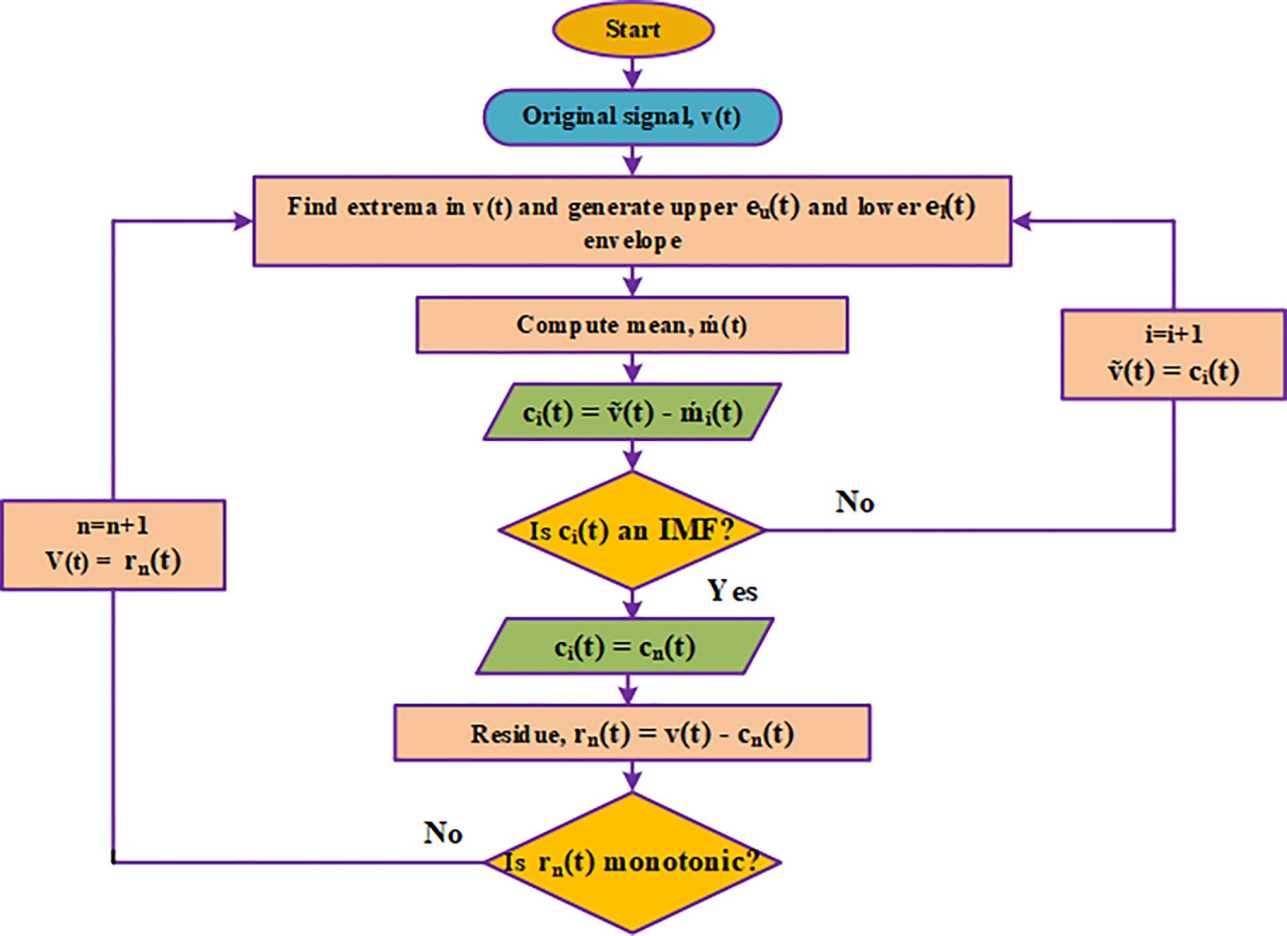
Flowchart of an EMD algorithm.

Two requirements must be met in order for a signal to qualify as an IMF: (i) The difference between the number of zero crossings and extrema is no more than one. (ii) The mean of the envelopes linking the local maxima and minima, respectively, is close to zero. Every IMF can therefore be thought of as a narrow-band signal reflecting a variable temporal scale inherent to the data, which is a crucial characteristic that sets EMD apart from Fourier approaches. The Fig 7 represents the flowchart of an EMD algorithm.

The standard EMD can be calculated in several steps. In this, the first and foremost one is computing the local mean and the local mean can be calculated by taking the average of both the upper and lower envelopes [43]. The standard EMD algorithm is shown in Table 1.

**Table 1:** The standard EMD algorithm.

The Standard EMD algorithm:
1. Let ṽ(t)=v(t) (*v*(*t*) is the original signal)
2. Identify all local maxima and minima of ṽ(t)
3. Find a lower envelope of *e*_*l*_(t) that interpolates all local minima
4. Find an upper envelope of *e*_*u*_(t) that interpolates all local maxima
5. Calculate the local mean, $ḿ(t)=\left(e_{l}(t)+e_{u}(t)\right)/2$
6. Subtract the local mean from ṽ(t), $c_{i}(t)=ṽ(t)-ḿ(t)$ (*i* is an order of IMF)
7. Let $ṽ(t)=c_{i}(t)$ and go to step 2 until *c*_*i*_(*t*) becomes an IMF

### 3.3 Features of EEG signals

For a given sequence g(n) = {g(1), g(2),…,g(N)}, where N is the length of the signal.

#### 3.3.1 Energy

When determining left-right MI EEG, energy is an important metric and can be calculated using the following formula.


$$E=\sum_{n=0}^{N-1}{\left|G_{t}(n)\right|}^{2}$$ (3.1)

#### 3.3.2 Autoregressive (AR) coefficients

AR models have been demonstrated to be a beneficial feature in MI recognition tasks and have been widely used in BCI research. The time-varying properties of signals are represented by the AR coefficients. In addition, AR is very good at modelling the EEG as filtered white noise with appropriate energy bands, which makes it perfect for EEG signal analysis. The following is the equation for the AR coefficient:

$$G(t)=\sum_{i=0}^{p}a(i)F(t-i)+e(t)$$ (3.2)

Where e(t) is the added white noise. The correct AR model order is set to seven.

#### 3.3.3 Fuzzy approximate entropy

The fuzzy approximation entropy (FAP) can be obtained using the procedures listed below.

A) From a given signal f(i), the structure $F_{i}^{m}$ vector

$$f_{i}^{m}=\{f(i),f(i+1),\ldots ,f(i+m‐1)\}‐f_{0}(i)\;i=1,2,\ldots ,N‐m+1$$ (3.3)

where,

$$f_{0}(i)=\frac{1}{m}\sum_{j=0}^{m-1}f(i+j)$$ (3.4)

B) Using the tolerance factor r, a fuzzy membership function has been established in order to calculate the maximal distance $d_{ij}^{m}$.


$$D_{ij}^{m}=exp(‐\frac{d_{ij}^{2}}{r})$$ (3.5)

C) The fuzzy approximate entropy is defined as follows:

$$FAP(m,r,N)=\Phi^{m}(r)‐\Phi^{m+1}(r)$$ (3.6)

Where,

$$\Phi^{m}(r)={\left(N-m+1\right)}^{-1}\sum_{j=1,j\neq i}^{N-m+1}\;\ln\;C_{r}^{m}(i)$$ (3.7)

Where,

$$C_{r}^{m}(i)={\left(N-m+1\right)}^{-1}$$ (3.8)

#### 3.3.4 Peak-to-peak amplitude

The difference between the signal’s maximum and minimum values is the peak-to-peak amplitude.


Peak‐to‐peakamplitude=max(F)–min(F) (3.9)

#### 3.3.5 Skewness

The asymmetry of a real-valued random variable’s probability distribution is measured by its skewness. Usually, the sample skewness is computed as follows:

$$Skewness=\frac{\sum_{i=1}^{N}{\left(f^{i}-ḟ\right)}^{3}}{N.s^{3}}$$ (3.10)

where sample mean and standard deviation is denoted as ḟ and s.

#### 3.3.6 Kurtosis

The probability distribution of a real-valued random variable’s is measured by kurtosis. One way to calculate sample kurtosis is to:

Kurtosis=Skewness–3 (3.11)

These features are calculated for each epoch (segments of EEG data corresponding to an event of interest) and are used to represent the characteristics of the EEG signal. The features are then concatenated into a feature vector for each epoch.

### 3.4 Convolutional neural network

**Fig 8:**
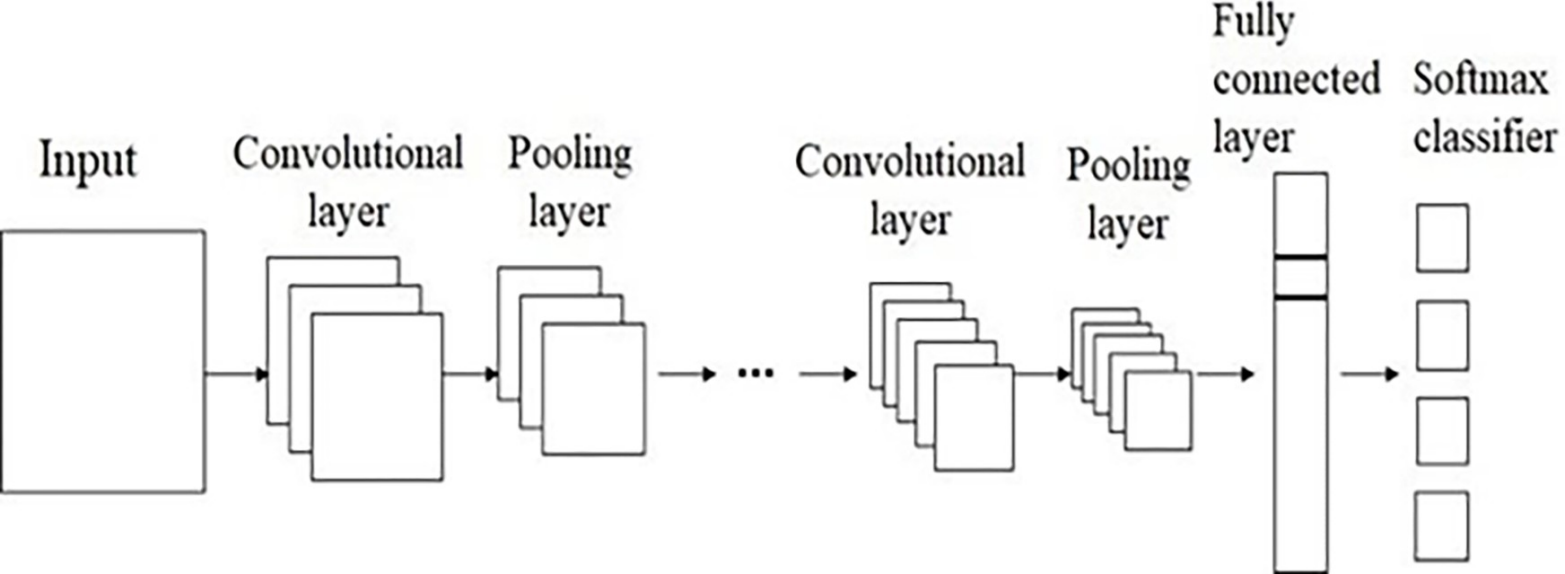
Basic block diagram of convolutional neural network.

Machine learning is a rapidly developing field, and deep learning is a crucial component of it. Deep learning has steadily emerged as a pioneer in several domains, drawing the interest of many researchers. CNNs are a popular neural network model in deep learning with several applications in different fields. Additionally, appropriate research on BCI systems is progressing in the intervening period. The Fig 8 illustrates the basic block diagram of a convolutional neural network.

In the middle of the network construction, several convolutional and pooling layers can be joined, followed by fully connected layers. The convolutional layer, which is at the core of CNN, is primarily responsible for extracting features from the input signal. It is essential that the convolutional layer be able to apply the necessary convolutional operations to the input signal. Convolutional kernels, sometimes referred to as filters, can be found in several instances inside a single convolutional layer. It is possible to modify the convolution kernels’ weight parameters and bias while the neural network is being trained. Convolutional operations can produce feature mappings from the input to the output by applying the concepts of matrix multiplication. It is assumed that the neural element is located at (m, n) in the feature map that the kth convolution kernel produces. Eq (4) shows the output.

$$y_{m,n}=f(w{\left(i\right)}_{k}*I(m,n)+b)$$ (3.12)

where f is the nerve element’s activation function, w(i)k is the kth convolution kernel of the i_th_ layer, b is the bias, and I(m, n) is the input data. Its frequent form includes tanch, rectified linear unit (ReLU) and sigmoid.


$$tanch:\;f(x)=\frac{1-e^{-2x}}{1+e^{-2x}}$$ (3.13)

$$sigmoid:\;f(x)=\frac{1}{1+e^{-x}}$$ (3.14)

ReLU:f(x)=max(0,x) (3.15)

On the other hand, more specific characteristics can be extracted through additional connections to tighter layers. In order to extract more complex abstract features from the signal as much as possible, it is possible to combine and superimpose many convolutional layers to constantly extract features from high-dimensional input data. The convolution layer processes actions like padding and stride in addition to the convolution operation, and the corresponding computational process is more complicated. The technique of lowering the input data’s length and breadth dimensions is called pooling, sometimes referred to as the sampling layer. The pooling layer can extract local information by performing a down sampling operation after the convolutional layer. The pooling layer makes it possible to reduce the number of network parameters, which also reduces the model’s complexity and computational load. In addition, this can help the network model’s overfitting issue and be incredibly resilient to slight data inaccuracies. Two main pooling operations are present in the pooling layer. By using maximum pooling, the biggest element value of the convolution kernel in the target region determines the feature value of the region. The convolution layer’s tiny flaws are optimized, and more local features are highlighted by the maximum pooling procedure. Similarly, by averaging pooling, the average value of its elements determines the feature value of the target region. The average pooling method not only preserves more adjacency information but also reduces the region error brought on by the convolution kernel’s size. In Eqs 3.16 and 3.17, the maximum and average pooling expressions are displayed. We suppose that the pooling kernel has a size of (N * N).

Max Pooling:

$$f(x)=max(x_{\left[m,m+N],[n,n+N\right]})$$ (3.16)

Average Pooling:

$$f(x)=\frac{1}{N*N}\sum_{m=m_{1},n=n_{1}}^{m=m_{1+N},n=n_{1+N}}x_{m,n}$$ (3.17)

In most cases, the fully linked layer is regarded as the last element in the CNN structure. When the feature space transformation travels through the fully linked layer, which integrates the previously extracted features for use in matrix multiplication, it transforms the feature maps created by the intermediate layers into vector format. The fully connected layer is in charge of converting the spatial high-dimensional information that the CNN has already retrieved and using non-linear mapping to wrap up the entire learning process.

The employment of design concepts including weight sharing, non-linear mapping of fully connected layers, sampling of pooling layers, sparse connectivity of convolutional layers, and sampling of pooling layers is primarily responsible for the CNN network model’s superior robustness and excellent generalization performance. For efficient feature extraction and classification recognition, a significant number of samples must be processed and examined in classical recognition research. Convolutional operations and other related operations are used to automatically train CNN models based on the inherent properties of the signal. CNN’s network model structure offers good interpretability in addition to excellent feature extraction effects.

## 4. Proposed EMD-PCNN method

The initial data is reduced by the first IMF,

$$r(t)=v(t)-c_{1}(t)$$ (4.1)

and the process is repeated on the residue *r(t)* until it stabilises or has stopped oscillating; this process, known as sifting, is guided by a properly defined stopping criterion [28]. The signal v(t) is then calculated by,

$$v(t)=\sum_{i=0}^{M}c_{i}(t)+r(t)$$ (4.2)

Where *c*_*i*_(*t*), *i* = 1,…, M are the IMF’s and *r*(*t*) is the remaining residue. By using the EMD, the above features are extracted such as energy, AR coefficients, fuzzy approximate entropy, peak-to-peak, skewness, kurtosis.

For performance improvement, several CNN models and approaches have been used in various sectors. As the convolution process proceeds layer by layer, CNN is able to automatically extract rich information. While the end layers are focused on extracting global characteristics, initial layers extract local and spatial features. Simple shapes like borders and boundaries might be considered lower-level features, while complicated shapes and entire objects are represented by high-level features. Convolutional features can accomplish different abstractions of object information at each level, as demonstrated by the numerous researchers who have extracted convolutional features from various layers and fused them to increase performance. The layered architecture allows for the loss of some important elements, which can be used to enrich the final feature set. Performance is increased even if the method uses more resources and has extra features. Researchers have developed many methods for extracting and combining multilayer convolutional features for various domains.

A parallel convolutional neural network (CNN) is a type of neural network architecture designed to process input data in parallel across multiple computational units or devices. In traditional CNNs, convolutional layers are applied sequentially, meaning each layer waits for the previous layer to finish processing before it can begin its own computations. However, in parallel CNNs, multiple convolutional layers or operations can be executed simultaneously, leading to potentially faster processing times and improved efficiency. The key components and characteristics of a parallel CNN are the following: The Convolutional layer is one of the fundamental building blocks of a CNN. Each layer applies a set of learnable filters (kernels) to the input data to extract features. In a parallel CNN, multiple convolutional layers can operate concurrently, distributing the workload across different computational units.

Parallelism in a CNN can be achieved at various levels, including data parallelism and model parallelism. Data parallelism involves splitting the input data across multiple processing units and applying the same operations simultaneously. Model parallelism, on the other hand, partitions the neural network model itself across different devices, with each device responsible for computing a portion of the model’s operations. Parallel CNNs are often deployed on multi-GPU or multi-core systems to exploit parallel processing capabilities. Each GPU or core can independently process a subset of the data or model parameters, allowing for faster training and inference. Efficient communication and synchronization mechanisms are crucial in parallel CNNs to ensure that different processing units can exchange information and coordinate their computations effectively. This is particularly important in scenarios where data or model parameters need to be synchronized across different devices. Parallel CNNs offer scalability benefits, allowing them to handle larger datasets and more complex models by leveraging additional computational resources. This scalability is especially valuable in tasks such as image recognition, object detection, and natural language processing, where large amounts of data and computational power are often required.

Overall, parallel convolutional neural networks enable faster training and inference times, improved scalability, and efficient utilization of computational resources, making them well-suited for tasks that demand high computational performance and scalability.

The one-dimensional convolutional neural network (1D-CNN), which is the foundation of the MI-EEG BCI system discussed here, is distinguished by the CNN kernel’s ability to only slide over the elements of the input pattern’s single dimension in this case, time during convolution. The 1D-CNN specifically accepts as input a matrix of dimensions M × N, where M is the time window length that is taken into consideration and N is the number of EEG channels. Specifically, a matrix with dimensions M × N where M is the length of the time frame under consideration and N is the number of EEG channels is fed into the 1D-CNN. In this case, M = 640, or 160 × 4 steps, and N = 2, or two symmetrical channels have as input. A variable dimension Q × N kernel is used in each 1D convolutional layer; Q represents the temporal window that the filter covers, and N = 2 because the kernel does not slide over the channels.

**Fig 9:**
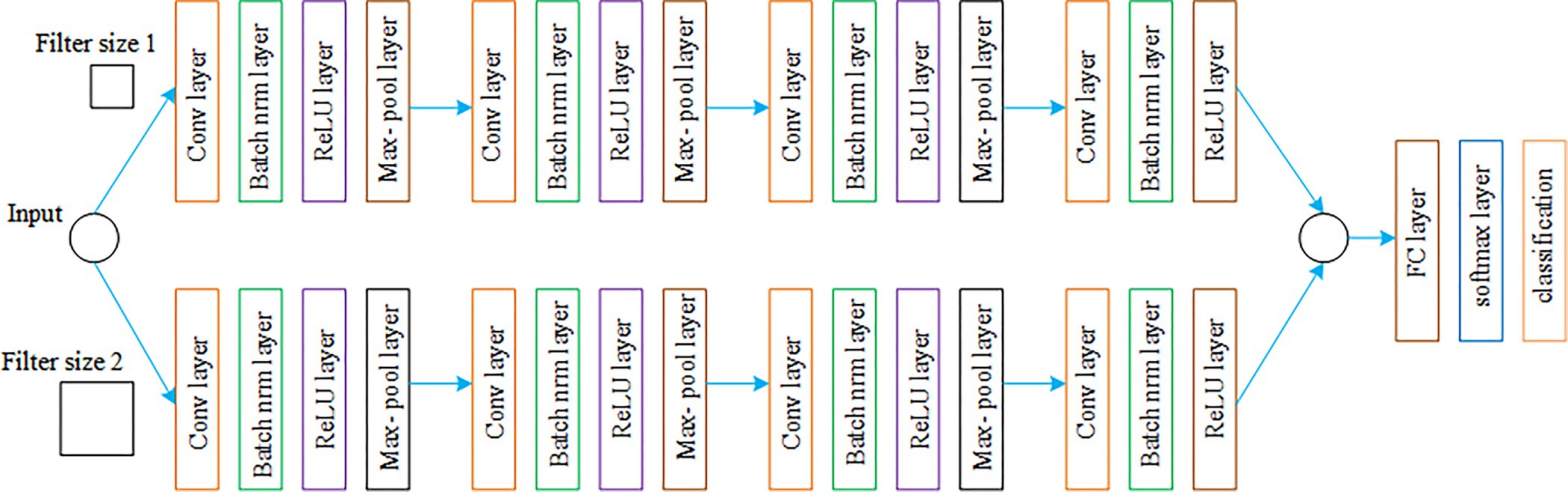
Block diagram of parallel convolutional neural network.

The 1D convolutional layer is represented mathematically as follows:

$$y_{r}=f(\sum_{q=1}^{Q}\sum_{n=1}^{N}w_{qn}X_{r+q,r+n}+b)$$ (4.3)

where y_r_ is the filter feature map of size R (R = M) output of unit r. The two-dimensional input component that overlaps the filter in the scenario when stride = 1 and padding is employed is denoted by x, the bias term is b, the activation function of the filter is f, and w is the convolutional filter’s connection weight. Following the convolution operation (R), we can use the following formula to get the dimension of the filter feature map:

The first convolutional layer (L1) employs padding to maintain the precise size of the input and output, and 32 filters of size 20 with a stride of 1. Batch normalization (BN) is used after the first convolutional layer. In order to improve network generalization, this entails normalizing the input to the following layer, which typically results in a significant boost in learning speed and noticeable regularization benefits. BN operates in a different way during testing and training. BN uses the whole batch (the collection of instances needed to calculate the loss and the gradient the learning algorithm uses) to normalize and zero-centre the input during training. The Fig 9 illustrates the block diagram of Parallel Convolutional Neural Network.

### 4.1 Network training and transfer learning

The categorical cross-entropy loss function served as the foundation for the neural network parameter optimization. The PCNN parameters were updated, and the category cross-entropy loss was minimized using the Adam optimization approach. In the deep learning literature, selecting the optimization algorithm is still up for discussion. We could have tested various optimization algorithms, but instead we have opted to stick with current one. Adam optimization is a stochastic gradient descent (SGD) approach that adjusts according to the gradient’s first- and second-order moments. It is based on a distinct learning rate for each parameter. Training employed a random order and a non-fixed number of epochs, where an epoch is a learning loop that uses all the examples once in a random order. To prevent overfitting, the validation early-stopping strategy was specifically applied. Through the use of transfer learning, a neural network can be trained to find general patterns and features in a large dataset (such as MI-EGG data from numerous individuals) and use those features to predict data on a new, similar dataset (such as data pertaining to a new individual) with the least amount of additional training. Using our method, we applied transfer learning to all subjects individually. A training set (70% of the data) and a test set (30% of the data) were separated from the dataset.

## 5. Results and analysis

Our proposed model EMD-PCNN is evaluated based on the two ways such as Qualitative and Quantitative.

### 5.1 Qualitative evaluation

The proposed EMD-PCNN method is analysed in a qualitative manner. The various evaluation parameters are used to evaluate the quality of the proposed technique. Such evaluation parameters are Specificity, Sensitivity, precision, and Accuracy. Specificity is used to identify the true negative of the proposed model, whereas sensitivity is used to identify the positive instances of the proposed method. Precision is used to trace or predict the true value of the proposed model. Accuracy is used to determine whether the classification model is correct overall and how often it acquires the correct value. Precision and accuracy are independent of each other. These evaluation parameters are analysed using the confusion matrix, which is described briefly in the following section.

#### 5.1.1 Confusion matrix

**Fig 10:**
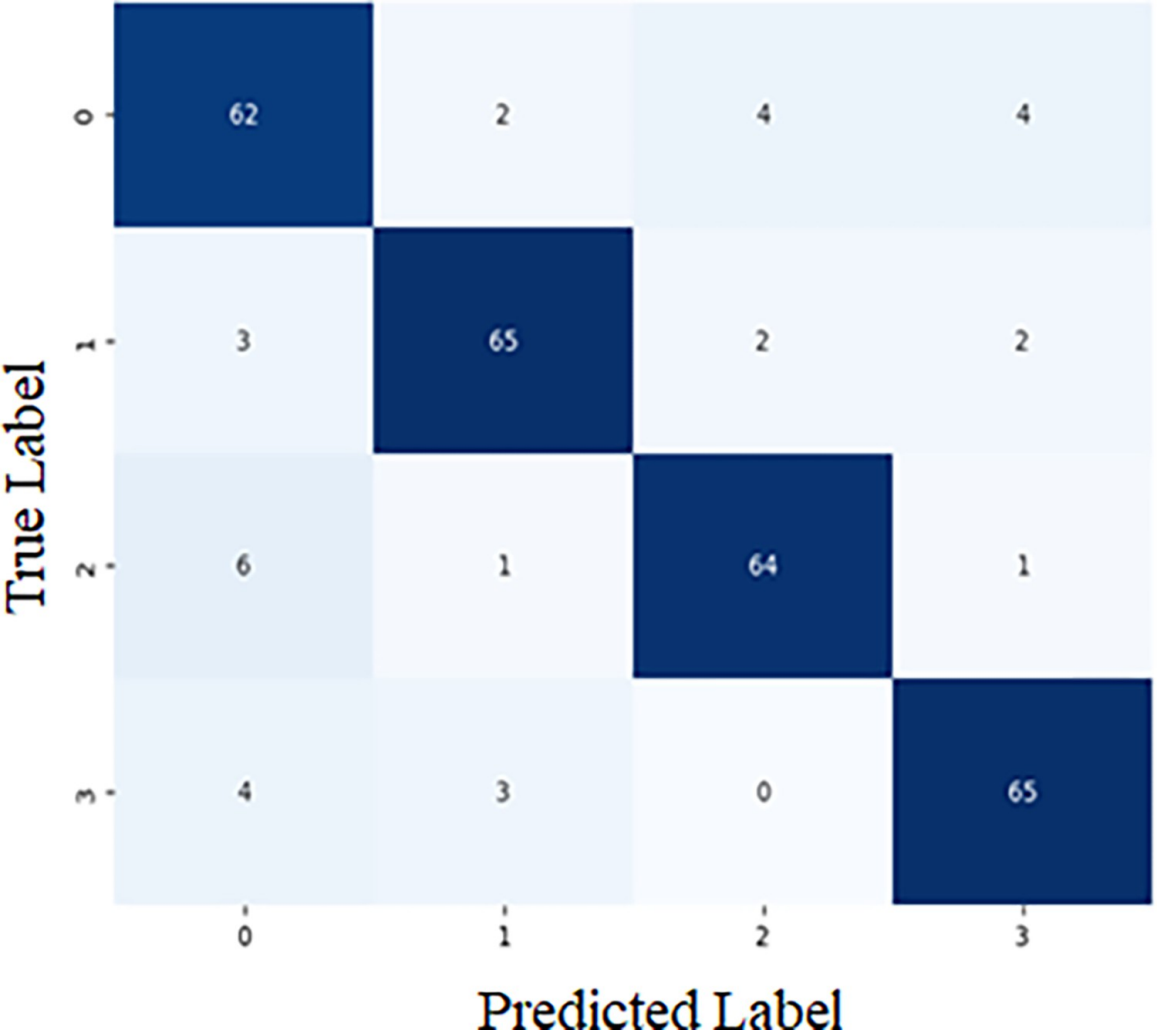
Confusion matrix of the proposed EMD-PCNN method.

A confusion matrix or an error matrix is used in various fields, especially in machine learning, to identify or solve the problem of the classification phase statistically. The algorithm performance is visualised in both the supervised and unsupervised, it is called the matching matrix in simple terms. In the confusion matrix, there are two classes available: one is the actual class, and the other is the predicted class. The predicted class is represented by each row of the matrix, and the actual class is represented by each column of the matrix, or vice versa. The Fig 10 illustrates the confusion matrix for the proposed EMD-PCNN method.

#### 5.1.2 Evaluation parameters

**Fig 11:**
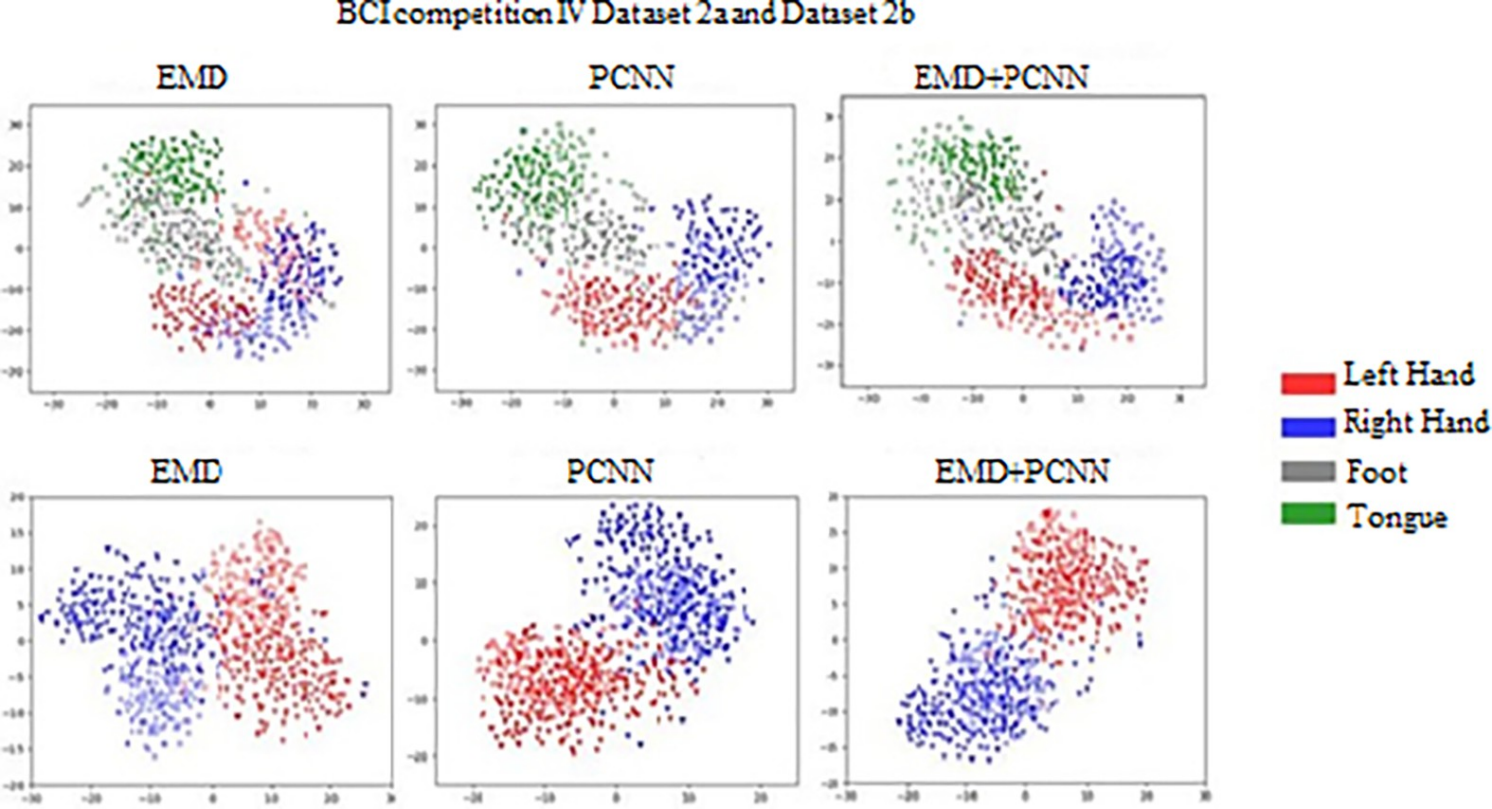
T-SNE visualization of the proposed method EMD-PCNN.

From the confusion matrix, we can calculate different evaluation metrics such as Specificity, Sensitivity, Precision Accuracy, Recall and F1-score for the validation of the model. The evaluation parameter calculation is done based on the following Eqs 5.1, 5.2, 5.3, 5.4, 5.5, and 5.6. Such evaluation metrics are used to assess the classification efficiency of the proposed EMD-PCNN technique. The Table 2 illustrates the hyper parameter setting of our proposed method EMD-PCNN. Table 3 illustrates the Subject-specific classification results for each subject for the BCI IV dataset (in %) and Table 4 Performance results of the Proposed method (in %). The T-SNE visualization of the proposed method EMD-PCNN is illustrated in Fig 11.

**Table 2:** Hyper parameter setting of our proposed method EMD-PCNN.

Hyperparameter	Settings
Pooling	Max pooling, Average pooling
Dropout	0.5
Learning Rate	0.001
Batch Size	32
Activation Function	ReLU
Regularization	L2 regularization
Optimizer	Adam

**Table 3:** Subject-specific classification results for each subject for the BCI IV dataset (in %).

Subjects	Tang et al. [44]	Fang et al. [45]	Syed Umar Amin et al. [46]	Dai et al. [47]	Xunguang et al. [48]	Pfurtscheller et al. [49]	Yu Xie et al. [50]	Proposed Method (BCI IV 2a)	Proposed Method (BCI IV 2b)
Subject 1	86.56	87.5	90.21	90.07	98	98.82	98.61	99.82	99.61
Subject 2	62.29	65.28	63.4	80.28	98	98.64	98.29	99.36	98.45
Subject 3	89.86	90.28	89.35	97.08	95	96.92	97.99	98.44	99.63
Subject 4	65.61	66.67	71.16	89.66	96	96.50	97.1	99.82	99.67
Subject 5	55.19	62.5	62.82	97.04	86	92.75	96.72	98.56	99.54
Subject 6	48.47	45.49	47.66	87.04	92	91.84	96.37	98.99	99.37
Subject 7	86.07	89.58	90.86	92.14	94	95.07	97.69	98.81	99.84
Subject 8	78.41	83.33	83.72	98.51	93	95.25	97.22	98.91	98.86
Subject 9	76.05	79.51	82.32	92.31	98	99.23	98.51	99.33	99.67
Average	72.05	74.46	75.72	91.57	94	96.13	97.61	99.01	99.39

**Table 4:** Performance results of the proposed method (in %).

Metrics	Dataset 1 (BCI IV 2b)	Dataset 2 (BCI IV 2a)
Specificity	97.35	96.88
Sensitivity	96.90	95.59
Precision	98.0	97.28
Accuracy	99.39	99.01
Recall	96.30	94.75
F1-Score	97.68	96.50

5.1.2.1 Specificity.


$$Specificity=\frac{TN}{TN+FP}$$ (5.1)

5.1.2.2 Sensitivity.


$$Sensitivity=\frac{TP}{TP+FN}$$ (5.2)

5.1.2.3 Precision.


$$Precision=\frac{TP}{TP+FP}$$ (5.3)

5.1.2.4 Accuracy.


$$Accuracy=\frac{TP+TN}{TP+TN+FP+FN}$$ (5.4)

5.1.2.5 Recall.


$$Recall=\frac{TP}{TP+FN}$$ (5.5)

5.1.2.5 F1- score.


$$F1‐Score=\frac{2\;X\;Precision\;X\;Recall}{Precision+Recall}$$ (5.6)

### 5.2 Quantitative evaluation

**Fig 12:**
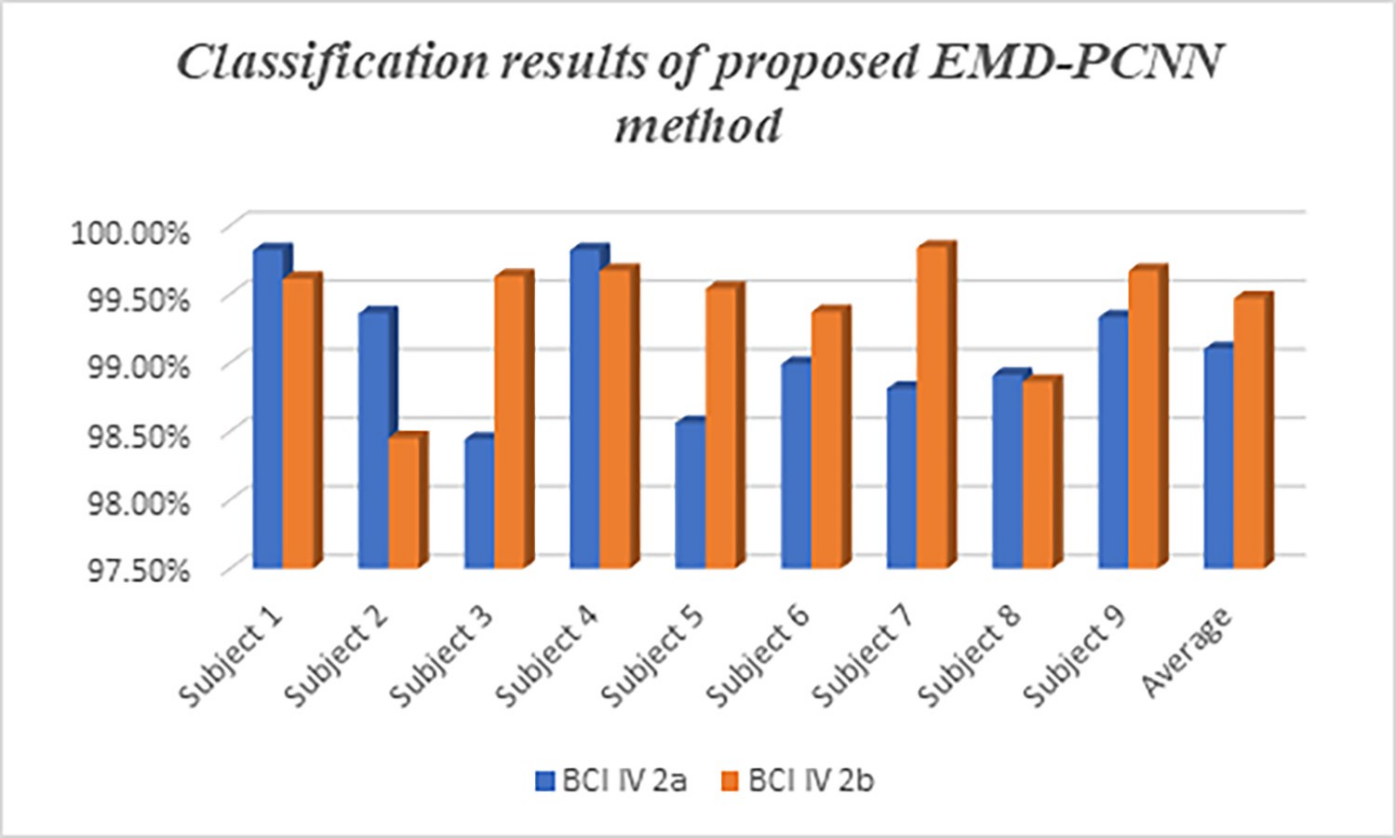
Subject classification results obtained for the proposed EMD-PCNN method.

The quantitative evaluation of the proposed method is discussed in Table 5 which describes the description of the proposed method and the existing one with the classification accuracy. Ang et al. (2012) employed a filter bank common spatial pattern (CSP) method achieving a 68% accuracy, while Tabar et al. (2016) used a 1D CNN with a stacked autoencoder (SAE) reaching 70%. Lawhern et al. (2018) implemented a CNN with depth and separable convolutions, yielding a 60% accuracy. Schirrmeister et al. (2017) introduced cropped training in a CNN architecture, improving accuracy to 72%. Sakhavi et al. (2018) combined temporal features with filter bank CSP and CNN, resulting in a 74.4% accuracy. Kevric and Subasi (2017) achieved a notable 92.8% accuracy with a KNN model using wavelet packet decomposition. In 2021, Mandeep Kaur Ghumman and Satvir Singh applied a support vector machine with CSP, achieving a 66.4% accuracy, while Rahul Sharma et al. (2022) used a multi-layer perceptron model that attained a high 92% accuracy. Arunabha M. Roy’s 2022 studies introduced a multi-scale CNN and a transfer learning-based multi-scale feature fused CNN (MSFFCNN), reaching accuracies of 93.74% and 94.06%, respectively. In 2023, Yu Xie et al. applied a CNN with continuous wavelet transform, achieving an impressive 97.61% accuracy. The highest accuracy, 99.39%, is attributed to the proposed method combining a PCNN with Empirical Mode Decomposition (EMD), showcasing the significant advancements and potential of hybrid deep learning approaches in EEG classification. In Fig 12, the subject classification results obtained by using BCI competition IV dataset for the proposed method has been illustrated.

**Table 5:** Classification results obtained statistically from the BCI dataset.

Method	Description	Classification Accuracy
Ang et al., [51], 2012	Filter bank CSP	68.0%
Tabar et al., [52], 2016	1D CNN with SAE	70.0%
Lawhern et al., [53], 2018	CNN with depth and separable	60.0%
Schirrmeister et al. [54], 2017	CNN with cropped training	72.0%
Sakhavi et al. [55], 2018	Temporal features with FBCSP and CNN	74.4%
Kevric and Subasi, 2017 [56]	KNN with Wavelet Packet Decomposition	92.8%
Mandeep Kaur Ghumman and Satvir Singh, [57], 2021	Support Vector Machine with CSP	66.4%
Rahul sharma et al. [58], 2022	Multi-layer perceptron model	92%
Arunabha M. Roy [59], 2022	Multi-scale convolutional neural network	93.74%
Arunabha M. Roy [60], 2022	Transfer learning (TL)-based multi-scale feature fused CNN (MSFFCNN)	94.06%
Yu Xie et al., [61], 2023	CNN with continuous wavelet Transform	97.61%
Proposed EMD-PCNN	PCNN with Empirical Mode Decomposition	99.39%

## 6. Conclusion

Motor imagery EEG signals hold significant promise for brain-computer interface (BCI) applications, enabling users to control external devices through cognitive processes. However, accurate classification of these signals remains a challenge due to their non-stationary nature and complex spectral characteristics. In this paper, we propose a novel approach that combines empirical mode decomposition (EMD) and parallel convolutional neural networks (PCNN) to address these challenges. By decomposing EEG signals into intrinsic mode functions (IMFs) using EMD and employing PCNN for feature classification, we aim to enhance classification accuracy and robustness. Our methodology consists of two main stages: feature extraction and classification. In the feature extraction stage, EEG signals are decomposed into IMFs using EMD, which effectively captures their non-stationary characteristics. Subsequently, these IMFs are used as input features for the PCNN classifier. The PCNN architecture is designed to operate in parallel, enabling efficient processing of multi-dimensional input data and extraction of discriminative features. We evaluate the proposed EMD-PCNN approach using the BCI Competition IV datasets 2a and 2b, which contain motor imagery EEG recordings from multiple subjects performing different tasks. We compare the classification performance of our method against existing approaches, including traditional machine learning algorithms and deep learning models. Our experimental results demonstrate that the EMD-PCNN method outperforms existing techniques in terms of classification accuracy. Specifically, we achieve a classification accuracy of 99.1% for four classes and 99.39% for two classes, surpassing the performance of state-of-the-art methods. Additionally, qualitative analysis using confusion matrices provides insights into the effectiveness of our approach in accurately distinguishing between different motor imagery tasks. Furthermore, quantitative evaluation metrics such as specificity, sensitivity, precision, accuracy, recall, and F1-score further validate the superiority of our method. In conclusion, we have presented a novel approach for the classification of motor imagery EEG signals using empirical mode decomposition and parallel convolutional neural networks. Our method demonstrates significant improvements in classification accuracy compared to existing techniques, effectively addressing the non-stationary nature of EEG signals. The proposed EMD-PCNN framework holds promise for enhancing the performance of BCI systems, paving the way for more reliable and efficient brain-computer interfaces. Future research directions may involve exploring additional feature extraction techniques and optimizing the PCNN architecture for real-time applications.

### 6.1 Future scope

Future studies will concentrate on enhancing deep learning algorithms to improve the classification of motor imagery EEG data and produce a more dependable brain-computer interface. We believe that the results of this study will encourage other researchers to use neural networks to improve the ability to classify brain signals and in future, by using the proposed result an external application or devices will develop for severely disabled people.

## Supporting information

S1 Dataset (GDF)

S2 Dataset (GDF)

S3 Dataset (GDF)

S4 Dataset (GDF)
